# A Compendium of Urinary Biomarkers Indicative of Glomerular Podocytopathy

**DOI:** 10.1155/2013/782395

**Published:** 2013-11-13

**Authors:** Miroslav Sekulic, Simona Pichler Sekulic

**Affiliations:** ^1^Department of Laboratory Medicine and Pathology, University of Minnesota, 420 Delaware Street SE, MMC 609 Mayo D185, Minneapolis, MN 55455, USA; ^2^Division of Nephrology, Department of Medicine, Harvard Medical School, Massachusetts General Hospital, 149 13th Street, Boston, MA 02129, USA; ^3^University of Belgrade School of Medicine, Dr. Subotica 8, 11000 Belgrade, Serbia

## Abstract

It is well known that glomerular podocyte injury and loss are present in numerous nephropathies and that the pathophysiologic consecution of disease hinges upon the fate of the podocyte. While multiple factors play a hand in glomerulopathy progression, basic logic lends that if one monitors the podocyte's status, that may reflect the status of disease. Recent investigations have focused on what one can elucidate from the noninvasive collection of urine, and have proven that certain, specific biomarkers of podocytes can be readily identified via varying techniques. This paper has brought together all described urinary biomarkers of podocyte injury and is made to provide a concise summary of their utility and testing in laboratory and clinical theatres. While promising in the potential that they hold as tools for clinicians and investigators, the described biomarkers require further comprehensive vetting in the form of larger clinical trials and studies that would give their value true weight. These urinary biomarkers are put forth as novel indicators of glomerular disease presence, disease progression, and therapeutic efficacy that in some cases may be more advantageous than the established parameters/measures currently used in practice.

## 1. Introduction

The glomerulus is the functional unit of the kidney that creates from the afferent blood flow an ultrafiltrate, which passes through the remainder of the nephron for further modification. The glomerulus can be compared to a selective filter in that it discerns based on size and charge what passes on through to become the formed ultrafiltrate. This selectivity is intrinsic to the glomerular filtration apparatus and is dictated by the structural integrity of the three main components of which it is made: the capillary endothelium, the glomerular basement membrane (GBM), and the visceral epithelium overlying the GBM. Material that navigates through the glomerular filter must pass through these three components in that order to attain passage from the vascular capillary side into Bowman's space. The visceral epithelial layer of the glomerulus is made up of podocytes: terminally differentiated cells that have formed foot processes that encircle the GBM. The foot processes interdigitate between one another, with the space between each laying the slit diaphragm: described as a zipper-like interaction of membrane proteins between adjacent podocyte foot processes [1]. The slit diaphragm is considered to be the size limiting component of the glomerular filtration apparatus: permitting material approximately under the size of albumin, while limiting the passage of molecules the size of serum albumin—70 kilodaltons (kDa)—and larger [2]. The visceral epithelial layer is continuous with the parietal epithelial layer—the layer that constitutes Bowman's capsule—and there are phenotypic characteristics displayed by the two that allow for differentiation [3, 4]. A novel concept poses that there may be additional layers to designate within the glomerular filtration apparatus: an endothelial surface layer and a subpodocyte space, which gives new insight on the generally accepted structure of the glomerulus [5]. Further, more detailed discussion of the components of the glomerulus will be limited in this paper; however, there are numerous reviews that pour over the details of each [6–17]. 

**Figure 1:**
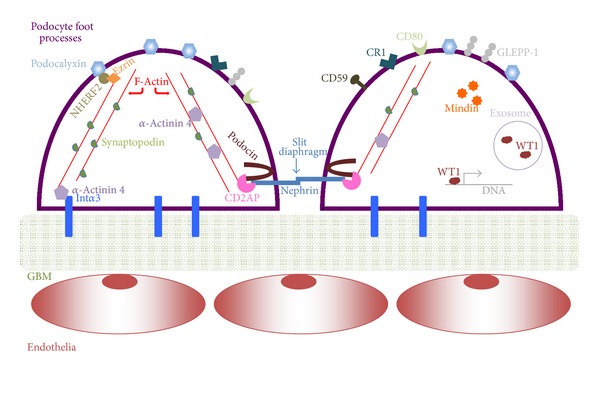
The protein biomarkers discussed in the paper are highlighted in this figure to show their cellular localization and the protein-protein associations that occur in glomerular podocyte foot processes. The podocyte is situated upon the glomerular basement membrane (GBM) and is anchored to it in part by alpha 3 integrins (Int*α*3). The slit diaphragm is illustrated with nephrin from adjacent foot processes interacting and forming a “zipper-like” structure. One can appreciate how the actin cytoskeleton (composed of F-actin) brings together the apical, lateral, and basal surfaces of the foot process: membrane proteins (podocalyxin, nephrin, alpha 3 integrin, etc.) via adaptor proteins (CD2AP, *α*-actinin 4, etc.) interact with actin, and as such they can modulate it. Wilms tumor protein 1 (WT1) has been identified as a transcription factor and is also localized within exosomes. Mindin has been localized within glomeruli, specifically to podocytes, and in cell culture found to be secreted into culture medium. Further details of each protein can be found in their respective sections within the paper.

As described before, the visceral epithelial layer of the glomerulus is made up of podocytes. The podocyte cell itself is comprised of a cell body, major processes which are large projections from the cell body, and long foot processes that adhere to the GBM. Three surfaces of a foot process can be described as apical, lateral, and basal, and while each surface is characterized by distinctive functional and structural roles, they are intertwined through intracellular associations. The apical surface faces Bowman's space, and with anionic surface proteins such as podocalyxin and podoplanin localized on the podocyte cell membrane, it is partially responsible for the repulsion of anionic molecules passing through the glomerular filtration apparatus [18–20]. Also on the apical membrane there is a protein tyrosine kinase phosphatase receptor named glomerular epithelial protein 1 (GLEPP-1), which is noted to regulate podocyte structure and function [21]. This negative charge lining the membrane surface of podocytes is also considered to be responsible for the maintenance of separation between adjacent foot processes [22]. The lateral surface of the foot process is comprised of the slit diaphragm, which is made up of many proteins, most notably the transmembrane protein nephrin that has numerous intracellular associations with both structural and signaling roles for the podocyte [23]. In mature podocytes, the slit diaphragm is also the only interaction between adjacent foot processes, and loss of this normal cell-cell interaction can be noted in the diseased state [9]. The basal surface of the podocyte foot process is attached to the GBM by the way of integrins, dystroglycans, and tetraspanins [24, 25]. Of the integrins that relate the podocyte to the GBM, *α*3*β*1 integrins are the most numerous [16]. The highly specialized structure of the podocyte is essential for its ability to function as part of the glomerular filtering unit, and when its structural integrity is compromised, so is its ability to parse out what material from the blood may pass through to form the ultrafiltrate. Much of the structure of the podocyte is based on its actin cytoskeleton, whose numerous interactions with surface proteins such as podocalyxin and nephrin highlight the fact that though the surfaces of the foot processes may seem to have separate roles and constitutional make-up, they are in fact highly related.  Figure 1 illustrates the basic cellular organization of the podocyte foot process, with specific emphasis upon the localization and the protein-protein associations amongst the soon to be discussed biomarkers of podocytopathy.

Podocytes are responsible for maintaining normal glomerular filtration in a number of ways: both in mechanical and functional fashions. Via the slit diaphragm, the podocytes (as previously described) act as barrier to protein based upon size. By way of the negatively charged surface of the podocyte foot processes, they serve as a barrier based on charge thus impairing passage of anionic proteins/material [12]. As a pericyte-like cell that encircles glomerular capillaries, a podocyte physically influences its vascular neighbors. Podocytes act as both a maintainer of glomerular capillary lumen patency and as a counteracting support against the intracapillary hydrostatic pressure (approximately 60 mmHg, a pressure that is greater than that found in most capillary networks) that would otherwise cause bulging [26]. It is notable in the pathogenesis of glomerulosclerosis that when podocytes are lost due to injury, the intracapillary pressure is no longer held in check by the podocyte counter-pressure, allowing the nude glomerular capillary to bulge and abut the parietal epithelium [10, 22]. Adhesions between the glomerular capillary and parietal epithelium are histologically evident in states when podocyte loss occurs [4]. In a functional manner, podocytes also lend to normal glomerular integrity by way of the elaboration of vascular endothelial growth factor-A (VEGF-A), which is necessary for the maintenance of the glomerular endothelium. VEGF-A has been considered to play a role in the pathogenesis of preeclampsia, with reduced expression of VEGF-A resulting in proteinuria and endotheliosis: both are pathognomonic renal findings in preeclampsia. The connection between preeclampsia and podocytes has been enlightened by findings that podocytes can be found in urine of women with preeclampsia and may be a diagnostic tool in diagnosing this gestational pathology [27]. Further glomerular capillary maintenance by podocytes is evident by their expression of angiopoietins (specifically Ang1) and the presence of Ang1 receptors (protein tyrosine kinase 2-Tie2) on glomerular capillary endothelium [28, 29]. The relationship between the podocyte and the underlying GBM is more than a static physical relationship. It has been shown that while both the glomerular endothelium and podocyte are responsible for production of the GBM in the developing kidney, there is a transition where podocytes resume responsibility and continue to add material to the GBM even at the stage of the adult glomerulus [30]. In addition, podocytes clear material that may deposit in the GBM, aiding in our understanding of how this filtering apparatus remains unclogged from debris over time [31]. Podocytes have also been shown to produce platelet-derived growth factor (PDGF), which has trophic properties for neighboring mesangial cells [32]. There is also evidence that podocytes may have a hand in immune response, with localization and isolation of CD80 and complement receptor 1 (CR1) from podocytes [33, 34]. The podocyte plays not simply a static, mechanical role, but it also has paracrine activity through elaboration of factors necessary for maintaining glomerular homeostasis and may have an active role in immune mediated functions within the glomerulus.

**Figure 2:**
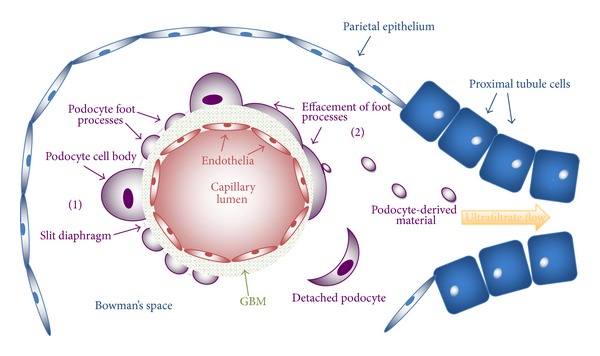
A simplified glomerulus and initial segment of the nephron are depicted; mesangia and the interweaved, capillary tuft structure are not shown to focus upon a single capillary and its related glomerular filtration apparatus. Also not depicted is the continuity between the visceral and parietal epithelial layers of the glomerulus. A glomerular capillary is lined by the fenestrated endothelia, which is intimately associated with the glomerular basement membrane (GBM). Also associated with the GBM is the visceral epithelial layer consisting of podocytes; the podocyte is made up of a cell body and processes that allow it to wrap finger-like projects about glomerular capillaries. Interposed between podocyte foot processes is a space spanned by the slit diaphragm—a key structure in the limitation of particles (based on size) from passing into the ultrafiltrate. Material/fluid that passes through the endothelia, GBM, and podocyte/slit diaphragm (these three constitute the glomerular filtration apparatus) makes it into Bowman's space, and the fluid at this point is termed ultrafiltrate. The ultrafiltrate from here passes through the length of the nephron—with modification possible—and urinary tract. The podocytes that lay on the left side of the shown capillary (1) are shown in a normal, healthy state: foot processes exist with developed slit diaphragms in between. In contrast, the right side of the capillary exhibits podocytes of an injured phenotype (2) effacement is present (other manifestations of an injured podocyte are not depicted, but can be read about in the paper), and at times podocytes may shed material later to be found in urine. If the podocyte is injured to a certain degree, it may detach from the underlying GBM and be observed in collected urine. A GBM denuded of podocytes may develop and lend itself as a surface for adherence with the parietal epithelium.

There are numerous etiologies for which podocyte injury manifests as clinically evident disease: most notably as proteinuria. When injured, podocytes undergo histologically notable changes in structure that directly relate to a reduction of normal filtrating function. Most characteristically, podocytes can undergo a process termed effacement, where the normal cellular architecture of the foot process is altered and results in flattening and spreading of the foot process along the GBM and disruption of the slit diaphragm. Effacement can manifest whenever the following occurs: when the slit diaphragm and its related protein-protein interactions are disrupted, when there is interference in the normal association of the foot processes with the underlying GBM, when there is reorganization of the actin cytoskeleton and its related protein-protein interactions, or when there is disruption of the normal negatively charged apical surface of the foot processes [14]. Effacement can be accompanied by progressive changes in podocyte structure characterized by vacuolization, formation of pseudocysts, apical membrane microvillus formation, disruption of podocyte foot processes from the underlying GBM, and consequentially, in appropriate conditions, loss of podocytes [8, 35].  Figure 2 depicts a simplified view of the glomerular filtration apparatus and the consequences of podocyte injury.

Glomerular pathology that includes injury to podocytes has been classically diagnosed by the analysis of biomarkers related to renal dysfunction and structural damage: blood urea nitrogen (BUN), creatinine in urine and blood, measure of creatinine clearance, and qualitative/quantitative measures of proteinuria. Though these measures are generally cheap and noninvasive, the final diagnosis of any patient with a suspected glomerulopathy is based upon the histologic findings from a kidney biopsy: a procedure that is not without risk [36]. The integrity of the slit diaphragm—a zipper-like interaction of membrane proteins between adjacent podocyte foot processes—is necessary for the inhibition of abnormal passage of proteins which have the size of and larger than albumin into the ultrafiltrate, and once the slit diaphragm is disrupted by way of podocyte injury, one can note the states of proteinuria that can range from inconspicuous to that of a florid nephrotic state (>3 g/day protein in urine). It should be added that while proteinuria is commonly associated with podocyte/slit diaphragm disruption, injury to the GBM or the glomerular capillary endothelium also results in proteinuria and thus brings to attention the lack of specificity of proteinuria for podocyte damage [37]. The level of proteinuria has also been found to be limited as a measure of monitoring disease progression as well as being limited for the monitoring of disease response to therapy [38]. Quantification of proteinuria is, however, still recognized as a simple and useful measure of glomerular injury and has been shown to correlate with progression of chronic kidney disease and as an indicator for increased cardiovascular morbidity/mortality [39]. To be curt, this paper is not aiming to dispel the proven worth of utilizing proteinuria as an indicator of glomerular/podocyte injury, but instead it aims to highlight the advantages—or weaknesses—of novel markers of podocytopathy and the potential they hold for clinical utility. While the strengths, weaknesses, and proper usage of measuring protein in the urine for diagnostic purposes has been discussed in many papers [40–43], as with any instrument in medicine, there comes a time when better tools come to be and advance the ability of the clinician to more confidently care for a patient. The fact that proteinuria can be measured noninvasively and relatively cheap with a urine dipstick argues for the continuing usage of it as a biomarker of glomerular injury, at least until more advantageous tools fall into hand. With that, we shall proceed with the discussion of some potentially more efficacious indicators of podocyte injury.

**Figure 3:**
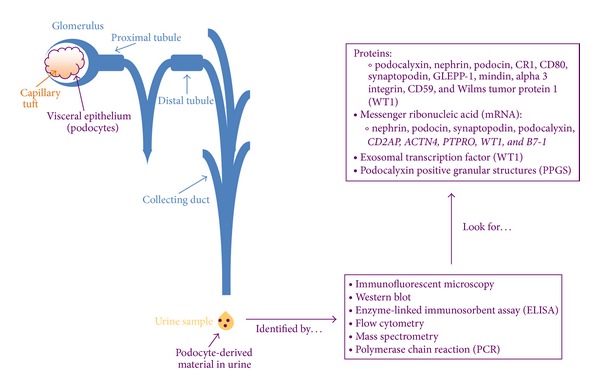
Podocytes envelop the capillary tuft of the glomerular body and are an integral part of the glomerular filtration apparatus. If podocytes are injured or lost via any number of mechanisms, podocyte specific material or whole podocytes themselves may traverse the length of the nephron and urinary tract to be collected/observed in the excreted urine product. With such material in hand, a number of noted techniques can be used to identify the podocyte derivations. More poignantly, investigations to date have described that such techniques can identify podocyte specific proteins, mRNA, an exosomal transcription factor, and podocalyxin positive granular structures.

Over the last two decades, investigations have shed light on the fact that podocytes are present in the urine in concurrence with a renal disease state and that podocyturia and biomarkers specific for podocytes themselves can relate to disease progression.  Figure 1 depicts a simplified view of the glomerular filtration apparatus and consequences of podocyte injury. It has also been noted that the level of podocyturia is a more sensitive indicator of disease activity than the level of proteinuria [44]. Notice should be taken of the fact that culture-viable podocytes can be isolated from the urine of healthy volunteers, so the specificity of podocyturia for glomerulopathies is not perfect [45]. In the urine of patients with certain glomerulopathies, evidence of podocyte injury can be found with the presence of viable podocytes (capable for the usage in primary cell culture), apoptotic podocytes, and podocyte derived exosomes and granular structures—all which have the possibility of being utilized as potential biomarkers of podocyte injury [46]. Urinary biomarkers that have been found to be specific for podocyte injury include proteins (podocalyxin, nephrin, podocin, CR1, CD80, synaptopodin, GLEPP-1, mindin, alpha 3 integrin, CD59, and Wilms tumor protein 1-WT1), podocyte specific messenger ribonucleic acid (mRNA) (nephrin, podocin, synaptopodin, podocalyxin, CD2-associated protein-*CD2AP*, *ACTN4*-encodes for *α*-actinin 4, *PTPRO*-encodes for GLEPP-1, *WT1*, and *B7-1*-encodes for CD80), an exosomal transcription factor (WT1), and podocalyxin positive granular structures (PPGS). Utilizing immunofluorescent staining, Western blot, enzyme-linked immunosorbent assay (ELISA), flow cytometry, and mass spectrometry, protein-based biomarkers are elucidated from urine samples of patients with glomerular disease, while polymerase chain reaction (PCR) is utilized to quantify the presence of podocyte specific mRNA.  Figure 3 illustrates the premise of the following urinary biomarkers in podocytopathies.

This review will describe the urinary biomarkers of podocyte injury that have been investigated to date: proteins, podocyte specific mRNA, an exosomal transcription factor, and PPGS. A brief characterization of each biomarker in normal and injured podocyte states shall be discussed along with an overview of how each has been examined as a urinary biomarker of podocyte injury.

## 2. Podocalyxin

Podocalyxin is an anionic, transmembrane protein that localizes to the apical membrane of glomerular podocytes. Aside from the kidney, podocalyxin is also localized in hematopoietic progenitor cells, vascular endothelium, and certain neurons [47–49]. Structurally, podocalyxin's extracellular portion consists of O-glycosylated, sialylated (providing the anionic charge), and N-glycosylated domains, along with a globular domain and a juxtamembrane stalk [50]. Intracellularly, podocalyxin has putative sites for phosphorylation along with a PDZ-binding domain. It is this PDZ domain that allows for podocalyxin's interaction with Na^+^/H^+^ exchanger regulatory factors 1 and 2 (NHERF1 and NHERF2) [51, 52]. Podocalyxin also interacts with ezrin—a protein that is found to be complex with NHERF2 and that binds actin—and thus relates podocalyxin to the cytoskeletal network that dictates podocyte structure [53]. Podocalyxin is also important in normal development of glomeruli, for knockout mice that do not express podocalyxin, and normal podocyte architecture is disrupted with histologic evidence of absence of foot processes and slit diaphragms [54]. It seems that in the glomerular podocyte, podocalyxin plays a role as the main contributor to the anionic surface of foot processes, and via its relation with the actin cytoskeleton it is involved in the normal formation and maintenance of podocyte structure.

Hara et al. first noted that podocalyxin in urine was related directly to podocyte injury in patients with IgA nephropathy and Henoch-Schönlein purpura nephritis [55, 56]. Many papers thereafter used their technique of immuno-staining against podocalyxin in urine samples to detect podocyte material. Hara had also noted that when podocalyxin was tested as a urinary biomarker for detecting the presence of varying glomerular diseases, it had a sensitivity of 88.4% and specificity of 100% [57]. When urine from patients with diabetes mellitus was examined, subjects with more severe disease (determined by severity of albuminuria) had significantly more podocalyxin positive podocytes in the urine than diabetic patients with less severe disease (defined as microalbuminuria equaling 20–200 *μ*g/min urine albumin excreted) and compared to healthy controls [58]. The same study also showed that treatment with trandolapril (an angiotensin converting enzyme inhibitor) reduced albuminuria and podocalyxin positive podocyturia in diabetic patients: a proof that podocalyxin and podocyturia are biomarkers that can be used to monitor therapeutic efficacy and disease progression. To further substantiate urinary podocalyxin's ability to reflect change in disease status with therapy, the adriamycin rat model of nephropathy induced increases proteinuria, podocyturia (detected via podocalyxin immunofluorescent staining), and foot process width. Treatment with 1,25-dihydroxyvitamin D_3_ reduced all mentioned parameters, showing that efficacy of therapy could be followed with the monitoring of urinary podocalyxin [59]. An investigation of adult patients with IgA nephropathy revealed that the severity of acute extracapillary abnormalities correlated with the level of urinary podocalyxin and that some patients with lower proteinuria and high urinary podocalyxin had poorer prognoses. The latter finding exhibits that in some cases proteinuria does not necessarily reflect the severity of disease but that the urinary level of podocalyxin does [60]. When podocytes, casts, and granules from the urine of children diagnosed with IgA nephropathy were immunofluorescently stained for podocalyxin, the degree of fluorescence directly correlated with the degree of glomerular epithelial injury as noted through renal biopsy histologic examination of the same patients [61]. These results would tend to allow one to believe that in children—and possibly adults—with IgA nephropathy, the degree of glomerular involvement and injury could be anticipated before renal biopsy through quantification of podocalyxin positive urine material, as well as a possible biomarker to follow disease progression. In a study examining patients diagnosed with focal segmental glomerular sclerosis (FSGS), it was shown that patients that were significant excreters of podocalyxin positive cells in the urine had a fall of serum creatinine in follow-up, lending to recovery [62]. Podocalyxin has been utilized as a urinary biomarker in studies examining podocyturia in preeclamptic women [63]. When podocalyxin was compared with podocin and nephrin in women with preeclampsia, it was found that podocin had superior diagnostic accuracy [64]. A separate study showed that levels of urinary podocalyxin were significantly greater in women with preeclampsia than normal pregnant women [65]. So where podocalyxin lies in the panel of podocyte biomarkers in the diagnosis of preeclampsia is yet to be decided. Urinary podocalyxin has also been detected—utilizing ELISA—in the urine of patients with acute kidney injury, and it was notable that urinary podocalyxin levels were greatest, whilst creatinine levels were recovering [66]. This group utilized the same assay to detect urinary podocalyxin in diabetic patients with glomerulopathy and found that there was a significant difference in control, healthy subjects and normoalbuminuric, diabetic patients [67]. This illustrates how a urinary biomarker specific for podocytes can detect disease otherwise passed over by the conventional monitoring of albuminuria. Early detection of podocyte lesions in diabetic glomerulopathy could give the clinician a chance to revert kidney disease by way of implementing appropriate therapeutic measures—a chance missed if one relied solely on the level of albuminuria. In order to create a more objective measure of urinary podocalyxin, a recent investigation used flow cytometry to detect the biomarker in the presence of glomerular disease [68].

Urinary podocalyxin mRNA expression has been illustrated to be significantly greater in patients with diabetic nephropathy compared to healthy controls, the level of mRNA increased with progression of disease, and the level of urinary podocalyxin mRNA positively correlated with urinary albumin excretion, BUN, and with serum creatinine [69]. Results from the same investigation showed that urinary podocalyxin mRNA negatively correlated with estimated glomerular filtration rate (eGFR). It should be noted that the quantitative analysis of urinary podocalyxin mRNA—and not just qualitative—should be recommended for clinical analysis, for podocalyxin mRNA has been shown to be expressed in urine of healthy, human volunteers [45]. All in all, it can be seen from the summary of studies utilizing podocalyxin as a urinary biomarker of podocyte injury—incurred through numerous different pathologies—that there is potential for its usage in the clinical setting to guide diagnosis and follow disease progression.

## 3. Nephrin

Nephrin is a 180 kDa transmembrane protein localized on the lateral surface of the podocyte foot process and is a major component of the slit diaphragm. Aside from podocytes, nephrin can be found in central nervous system tissue and pancreas, specifically in the *β*-cells of the islets of the Langerhans [70]. In the capillary loop stage of nephron development, nephrin can be localized at junctures of incipient podocyte foot processes, while nephrin mRNA expression can be detected in the S-shaped bodies of the developing nephron [71]. Nephrin—the product of the *NPHS1* gene—was discovered when mutations in *NPHS1* were found in patients with congenital nephrotic syndrome of the Finnish type [72]. Examination of kidney samples from patients with congenital nephrotic syndrome of the Finnish type shows thinning of the lamina densa layer of the GBM, yet otherwise insignificantly different from healthy, control kidneys [73]. An experiment utilizing a murine model, in which the *Nphs1* gene is inactivated, presents histologic evidence of podocyte foot process effacement and absence of a slit diaphragm, along with proteinuria and the immediate death soon after parturition [70]. Nephrin associates and colocalizes with podocin and the actin cytoskeleton, and the interaction of nephrin with the actin cytoskeleton is done so through an association with CD2AP [74, 75]. In addition, nephrin forms complexes—as proven through coimmunoprecipitation and pull-down assays—with cadherins, p120 catenin, and scaffolding proteins zonula occludens protein 1 (ZO-1), CD2AP, and calcium/calmodulin-dependent serine protein kinase (CASK) [76]. There is also a proof that nephrin and the slit diaphragm can be a source of intracellular signaling mechanisms in podocytes. This was made evident by the fact that nephrin expression triggers cellular activation via protein kinase p38 and the c-Jun N-terminal kinase in HEK293T cells [23]. Cellular activation was enhanced in the presence of podocin, and this would suggest that signaling involving the slit diaphragm requires normal interaction of nephrin and podocin. Nephrin's interaction with the actin cytoskeleton via CD2AP might also lend to the idea that nephrin's role in intracellular signaling may be transmissible to the cytoskeleton and result in structural changes. It can be appreciated that nephrin has a significant role as a constituent of the slit diaphragm, a role in maintaining normal podocyte foot process architecture, and an additional position in intra-podocyte signaling.

Urinary nephrin protein and mRNA have also been examined in patients with a number of glomerulopathies and hold potential as clinically useful biomarkers of podocyte injury. A study of patients with type 1 diabetes noted that while microalbuminuria was used for the early detection of kidney involvement in disease, advanced glomerulopathy was already present and presented that 30% of normoalbuminuric patients had nephrinuria; all healthy controls in the study were found to be negative for urinary nephrin [77]. The detection of urinary nephrin from type 2 diabetic patients determined to be normoalbuminuric also showed that urinary nephrin was a more sensitive marker of nephropathy in diabetes than the measure of urinary albumin [78]. Relatedly, observation of the FVB/NJ Akita mouse model of diabetic nephropathy noted that urinary nephrin could be detected before detectable albuminuria develops [79]. These data again highlight how a urinary biomarker specific for podocytes can detect disease otherwise passed over by the conventional monitoring of albuminuria. Investigators have recently shown that preeclamptic women had significantly higher levels of urinary nephrin than healthy pregnant women [65] and that there was a correlation between urinary nephrin levels and urinary protein, serum creatinine, and diastolic blood pressure in preeclamptic women [80]. Utilizing Heymann nephritis model, immunohistochemistry and PCR showed a decrease in both urinary nephrin protein and mRNA expression compared to healthy controls, and this decrease was noted before the onset of proteinuria was detected [81]. As part of the same study, it was shown that urinary nephrin could be quantified by Western blot and that healthy animals did not excrete detectable nephrin in their urine.

A second study utilizing a passive Heymann nephritis model found that nephrin protein and mRNA could be quantified from cells isolated from collected urine samples [46]. In a study using a streptozotocin model, it was noted that urinary cells could be stained against nephrin and that, in healthy controls, cells could not be detected in urine [82]. The same investigation showed that the cells collected from streptozotocin-treated rats were viable for primary culture. Aaltonen et al. noted similarly that urinary nephrin could be detected early in the streptozotocin rat model [83]. Podocyturia determined via nephrin staining did not significantly predict preeclampsia; however, the study noted that the urine of some women with severe disease was positive for podocyturia by nephrin staining [84]. Urinary nephrin mRNA levels increased after instigation of glomerular injury in models utilizing human diphtheria toxin receptor (hDTR) transgenic rats (expressing hDTR specifically on podocytes); however, on follow-up urine analysis, nephrin mRNA could not be detected [85]. Another study using hDTR transgenic rats showed that the podocin : nephrin mRNA ratio correlated with the renal histologic state [86]. The same study showed that human patients diagnosed with systemic lupus erythematosus (SLE) with associated glomerular disease had measurable levels of urinary nephrin mRNA. Urinary nephrin mRNA expression significantly correlated with the degree of proteinuria in patients with diabetic nephropathy, showing that nephrin mRNA in the urine could possibly be clinically used as a biomarker to follow glomerulopathy progression in diabetic patients [87]. In patients with lupus nephritis, urinary nephrin mRNA could be measured and was found to correlate with the level of proteinuria [88]. Urinary nephrin mRNA was found to correlate with the decline of renal function and degree of proteinuria in a study examining the progression of disease in a group of patients with diagnoses of diabetic glomerulosclerosis, IgA nephropathy, minimal change disease, and membranous nephropathy [89]. Further, supporting the strength of urinary nephrin mRNA in clinical utility, the level of mRNA was significantly greater in the urine of patients with glomerular kidney disease than healthy controls, and the level of urinary nephrin mRNA could differentiate between proliferative and nonproliferative disease [90]. Urinary nephrin mRNA levels could differentiate between preeclamptic women and healthy controls, as well as being used to differentiate between preeclamptic women and women with gestational diabetes [91]. Quantitative analysis of urinary nephrin mRNA—and not just qualitative—should be recommended for clinical analysis, since nephrin mRNA was expressed in urine of healthy, human volunteers [45]. It can be seen that the evidence for urinary nephrin (protein and mRNA) being used as a biomarker of podocyte injury is building, and in a number of different glomerulopathies could be of clinical use.

## 4. Podocin

Podocin is a 42 kDa membrane-associated protein that has a hairpin structure resulting with both N- and C-terminal ends located in the cytoplasm. The *NPHS2* gene—the product being podocin—is mutated in certain patients with autosomal recessive steroid resistant nephrotic syndrome, with disease manifesting early in childhood and progression to end-stage renal failure [92]. In development, podocin mRNA is first expressed in the S-shaped body of the incipient nephron, while podocin protein is first expressed in the capillary loop stage. In the mature kidney, podocin mRNA expression is only detectable in podocytes [93]. Localized to the lateral membrane of the podocyte foot process at the insertion of the slit diaphragm, podocin can be found in an oligomeric formation within lipid rafts and interacts with nephrin and CD2AP by way of a COOH-terminal domain [75]. Podocin plays an integral role in nephrin mediated cellular signaling, and thus it has a putative role in podocyte structure and function [23].

In a number of studies to date, podocin protein and mRNA have been shown to be quantifiable in the urine of both animal models of glomerulopathies and humans with renal disease. In a concerted effort to objectively measure a urinary podocyte biomarker, a recent study used mass spectrometry to detect podocin in urine samples of preeclamptic women [94]. It would be highly interesting to follow up with further studies detecting other discussed podocyte biomarkers with mass spectrometry. Nakatsue and colleagues have shown that while urinary nephrin was detectable by Western blot in diseased Heymann nephritis animals, podocin could not be detected in the urine of the same animals [81]. However, a different study from a second group had shown that podocin protein and mRNA could in fact be quantified from cells isolated from collected urine samples in passive Heymann nephritic rats [46]. Whether podocin protein can be quantified from urine of Heymann nephritis animals or the human equivalent of membranous nephropathy is still to be determined. As previously described in the podocalyxin section, in women, podocin had greater diagnostic accuracy compared to the utilization of podocalyxin and nephrin as biomarkers of podocytes in urine for determining the presence of preeclampsia [64]. A separate study noted similarly that urinary derived podocin mRNA was significantly elevated from preeclamptic women compared to healthy controls [91]. In a study using a streptozotocin model, it was noted that urinary cells could be stained against podocin and that in healthy controls cells couldnot be detected in urine [82]. Urinary podocin mRNA levels increased after the instigation of glomerular injury in models utilizing hDTR transgenic rats, and on follow-up urine analysis podocin mRNA could still be detected—in contrast to urinary nephrin mRNA which could not be detected [85]. Data from the same study showed that urinary podocin mRNA levels were found to positively correlate to the level of proteinuria, and human patients diagnosed with SLE with associated glomerular disease had measurable levels of urinary podocin mRNA. An investigation using hDTR transgenic rats illustrated that the podocin : nephrin mRNA ratio correlated with the renal pathohistologic state [86]. In patients with lupus nephritis, urinary podocin mRNA could be measured, and as the patients are followed over time, urinary podocin mRNA decreased along with a worsening glomerular filtration rate (GFR) [88]. This may be the evidence to support that urinary podocin mRNA could be a clinically useful biomarker for following disease progression in patients with lupus nephritis since it seems to trend along with the GFR, a measure of glomerular function. Urinary podocin mRNA expression was significantly greater in patients with diabetic nephropathy compared to healthy controls, the level of mRNA increased with progression of disease, and the level of urinary podocin mRNA positively correlated with levels of urinary albumin excretion, BUN, and with serum creatinine [69]. Urinary podocin mRNA could also be collected from some dogs with chronic kidney disease, but this study did not contain a healthy control group, so the significance of these findings is yet to be determined [95]. Urinary podocin mRNA was found to correlate with the decline of renal function in a study that examined the progression of disease in a group of patients with diagnoses of diabetic glomerulosclerosis, IgA nephropathy, minimal change disease, and membranous nephropathy [89]. Thus it can be seen that urinary podocin protein and mRNA have been found to be viable biomarkers of podocyte injury, though further investigations will be necessary to see their efficacy as a clinical tools.

## 5. Complement Receptor 1 (CR1)

Complement receptor 1 (CR1)—also known as CD35 or C3bR—is a 200 kDa membrane bound receptor that within the adult glomerulus is expressed only in podocytes [96, 97]. Outside of the glomerulus, CR1 expression is found in follicular dendritic cells, the Langerhans cells in the skin, the Küpffer cells, circulating erythrocytes, neutrophils, monocytes, and T-lymphocytes [98, 99]. CR1 protein is expressed in both fetal podocytes of the developing kidney and in the mature, adult kidney. In the fetal kidney, CR1 mRNA is detectable in immature podocytes in early stages of glomerular differentiation, specifically after vascularization of the developing glomerulus [33, 100]. By binding C3b—the active form of C3—CR1 regulates complement activation on the cell membrane by inhibiting activation of the complement pathway [101]. The specific role that CR1 plays on the surface of podocytes has yet to be fully elucidated, but one may assume that it may hold a protective role for podocytes from immune attack.

CR1 positive vesicles have been found by Western blot in the urine of healthy patients, and in comparison, urinary CR1 expression was decreased in patients with more advanced cases of SLE [102]. In a study by Hara, the diagnostic sensitivity and specificity of urinary CR1 for patients with an amalgam of glomerulopathies were determined to be 51.4% and 100%, respectively [57]. CR1 was also noted to have been localized to PPGS found in the urine of patients with active glomerulonephritis and nephrotic syndrome [103]. It has been shown that CR1 can be found in the urine of both healthy subjects and patients afflicted by glomerulopathies, but more work must be done in order to understand the expression of urinary CR1 in individual glomerular disease states. With the evidence in hand, it appears that CR1's utility in monitoring podocyte injury is lacking.

## 6. CD80

CD80 (also referred to as B7-1) is a 53 kDa membrane-associated protein that in the glomerulus is localized exclusively in podocytes, but it can also be found in renal tubules [104, 105]. It is better known for its role in the immune system as a costimulatory receptor involved in T-lymphocyte activation [104]. CD80 activation by puromycin in cultured podocytes is found to attenuate expression of nephrin and results in foot process effacement and retraction, providing insight on how CD80 can influence podocyte structure and function [106]. The ability of CD80 to regulate podocytes' filtering capacity is also shown when lipopolysaccharide is injected in mice, resulting in increased CD80 expression and proteinuria, and in mice that are knockouts for CD80, proteinuria does not occur [105]. Therefore, the immune stimulatory role of CD80 within the glomerular milieu would support the idea that it may modulate immune mediated injury to podocytes.

CD80 and its mRNA (*B7-1*) can be detected and measured in collected urine and may be potential biomarkers of podocyte injury. Urinary levels of CD80 in patients with relapsed minimal change disease were significantly greater when compared to those in patients with minimal change disease in remission, SLE (with and without proteinuria), other glomerulopathies (FSGS, membranoproliferative glomerulonephritis, IgA nephropathy, and membranous nephropathy), and healthy control patients [104]. Data from a second study by the same group showed that urinary CD80 was increased in patients with minimal change disease in relapse compared to patients with minimal change disease in remission or those with FSGS [34]. These results support that urinary CD80 could be used clinically to differentiate between patients with the relapsed minimal change disease and FSGS. Additionally, the level of urinary *B7-1* mRNA was found to be enhanced in patients with glomerular kidney disease compared to that of healthy subjects [90]. Promising data is presented for the utility of urinary CD80 for a biomarker of podocytopathy; however, much more work is needed to substantiate confidence in its clinical usage, and the fact that CD80 can also be derived from tubular epithelium reduces confidence in its specificity.

## 7. Synaptopodin

Synaptopodin (also referred to as podocyte protein 44-pp44) is an actin binding protein, and through this interaction it can modulate the podocyte actin cytoskeleton and resultant cellular function [107]. Synaptopodin is immunohistochemically expressed in podocyte foot processes and colocalizes with actin within the cytoplasm, and in the central nervous system synaptopodin mRNA expression is positive in the olfactory bulb, cerebral cortex, striatum, and hippocampus [108]. Synaptic plasticity in the hippocampus seems to be reliant on the activity of synaptopodin and its interaction with calcium stores [109]. During renal development, synaptopodin is first identified in the incipient glomerulus during the capillary loop stage—the stage at which foot processes formulate [110]. Synaptopodin—in the fully developed glomerulus—is exclusively expressed in podocytes [111]. More specifically, synaptopodin is a marker of the well-differentiated podocytes, as an investigation into the culturing of podocytes noted that synaptopodin expression is synonymous with the development of foot processes and a phenotype similar to that found *in vivo* [108]. It has been shown that synaptopodin is proteolytically downregulated by cathepsin L—a protease that is induced in the diseased state—resulting in actin reorganization, podocyte architecture change, and proteinuria [107, 112]. Thus the role that synaptopodin plays in maintaining the actin cytoskeleton is evident when its activity is attenuated, with subsequent podocyte dysfunction.

There are a number of studies that have examined the presence and utility of urinary synaptopodin protein and mRNA to determine podocyte damage. It was found in diabetic nephropathy patients that urinary synaptopodin mRNA significantly correlated with glomerular podocyte number observed in histologic examination, showing that urinary synaptopodin mRNA may be clinically applicative in monitoring disease progression in these patients [113]. Data from another study examining patients with diagnosed diabetic nephropathy presented that urinary synaptopodin mRNA correlated with the degree of proteinuria [87]. In patients with lupus nephritis, urinary synaptopodin mRNA was measured, and as the patients were followed over time, urinary synaptopodin mRNA decreased along with the GFR [88]. This may be the evidence to support that urinary synaptopodin mRNA could be a clinically useful biomarker for following disease progression in patients with lupus nephritis since it seems to trend along with the GFR. Urinary synaptopodin mRNA expression was significantly greater in patients with diabetic nephropathy compared to healthy controls, the level of mRNA increased with progression of disease, and the level of urinary synaptopodin mRNA positively correlated with the level of urinary albumin excretion and BUN [69]. Using a streptozotocin model of diabetic nephropathy, urinary cells could be stained against synaptopodin; however, in healthy controls, urinary cells stained for synaptopodin could not be detected [82]. The same group has also shown that synaptopodin protein and mRNA could be quantified from cells isolated from urine samples of passive Heymann nephritic rats [46]. Urinary synaptopodin mRNA was found not to correlate with the decline of renal function in a study examining the progression of disease in a group of patients with diagnoses of diabetic glomerulosclerosis, IgA nephropathy, minimal change disease, and membranous nephropathy [89]. In addition, synaptopodin mRNA was found in urinary podocytes of both FSGS patients and healthy humans [45]. Data from a study that included patients with a number of different glomerulopathies showed that urine sediment did not stain for synaptopodin [57]. Urinary synaptopodin is also noted to be a poor indicator of the preeclamptic state, as it was found to have diagnostic sensitivity of only 38% and specificity of 70% [114]. It is difficult to conclude from the collected studies if urinary synaptopodin protein or mRNA can be utilized regularly with confidence in clinical practice as biomarkers of podocyte injury.

## 8. Glomerular Epithelial Protein 1 (GLEPP-1)

GLEPP-1 (also referred to as protein tyrosine phosphatase receptor type-O—PTPRO) is an apical-membrane-associated protein tyrosine phosphatase found on glomerular podocytes [115, 116]. Central nervous system expression of *Ptpro* (encodes for GLEPP-1 protein) is localized to the olfactory bulb, developing neocortex, thalamus, and hippocampus [117]. In the developing kidney, GLEPP-1 protein expression is first detected during the S-shaped stage of the glomerular anlage, with increasing expression noted in podocytes through development and into maturity [118]. GLEPP-1′s importance in the maintenance of normal glomerular filtration has been shown in patients diagnosed with idiopathic nephrotic syndrome, steroid resistant subtype, that different mutations in *PTPRO* are to blame in resultant phenotypic changes of podocytes and massive proteinuria [119]. In *Ptpro* knockout mice, altered podocyte architecture can be observed with foot processes shorter and broader, and functionally these mice have reduced glomerular filtration function (25–50% lower GFR than wild-type) and a propensity for hypertension if the kidney is placed under increased functional stress (as exhibited in the animal model after nephrectomy) [21]. GLEPP-1 has an important role to play in normal podocyte function and the maintenance of glomerular integrity, and its activity as a phosphatase lends to the hypothesis for a putative role in intracellular signaling.

Urinary GLEPP-1 protein and *Ptpro* mRNA have been examined for usage as biomarkers of podocyte injury. Using a streptozotocin model of diabetic nephropathy, urinary cells could be stained against GLEPP-1; however, in healthy controls, urinary cells stained for GLEPP-1 could not be observed [82]. The same group also presented that GLEPP-1 protein and *Ptpro* mRNA could be quantified from cells isolated from urine samples of passive Heymann nephritic rats [46]. Utilizing a puromycin aminonucleoside (PAN) nephrosis model, *Ptpro* mRNA was found in the urine of rats after insult, but not in untreated, healthy rats [120]. It is obvious that there is a paucity of data relating to GLEPP-1's usage as a urinary biomarker of podocytopathy, and evaluation of its efficacy clinically is not possible at this time as thus.

## 9. Mindin

Mindin (also known as spondin-2) is a secreted protein that becomes a constituent of extracellular matrix, as it was first observed in the zebrafish floor plate [121]. Mindin mRNA is found in the brain, liver, spleen, placenta, lung, heart, kidney, muscle, and thymus, and in the central nervous system mindin has been implicated as a promoter of hippocampal neuron development [121, 122]. In the kidney, the immunohistochemistry shows that mindin localizes predominately to podocytes within the glomerulus and that secretion of mindin is appreciated in podocyte cell culture [123]. Inflammatory reactions are also mediated by mindin, and this has been illustrated in mindin knockout mice that are resistant to lipopolysaccharide-induced septic shock, and the same mice are not as capable in clearing bacterial infections *in vivo* [124]. Mindin is found to bind neutrophils through the involvement of integrins, and with localization in the glomerulus mindin may have a role as a recruiter of inflammatory cells in the pathogenesis of glomerular lesions [125].

While mindin has not previously been utilized as a biomarker of podocyte injury, a recent study by Murakoshi et al. has shed light upon mindin's histologic and urinary expression in diabetic nephropathy [123]. The study presented that mice fed on high calorie diets expressed augmented levels of glomerular mindin protein and mRNA than mice fed on a standard diet. As well, mice fed on high calorie diets were also found to have greater amount of mindin protein in their urine than their standard diet counterparts. In podocyte cell culture, glucose administration was found to accentuate mindin secretion. In humans with type II diabetes, the level of urinary mindin was greater than that measured in healthy controls, and the amount of urinary mindin increased in concert with progression of diabetic nephropathy disease. Thus, it can be appreciated that urinary mindin can function as a biomarker of podocyte injury in diabetic nephropathy; however, its general usage will be limited until further investigations can determine its efficacy in this and other glomerulopathies.

## 10. Alpha 3 Integrin (Int*α*3)

Int*α*3 is a heterodimeric, podocyte basal-membrane surface protein that is responsible for the attachment of the foot processes to the underlying GBM. During renal development, Int*α*3 is necessary for normal organogenesis, for in mice with a mutated Itga3 gene abnormal collecting duct, proximal tubule, and glomerular histology can be observed [24, 126]. The same mutated mice show a disrupted GBM and an immature phenotype of podocytes lacking formation of foot processes. Int*α*3 is also shown to be necessary for normal pulmonary organogenesis, with abnormal branching of bronchi being observed. In patients found to be homozygous for mutations in the gene encoding Int*α*3, basement membrane disruption was notable in numerous tissues and presented clinically with congenital nephrotic syndrome, interstitial lung disease, and epidermolysis bullosa [127]. Int*α*3 is an important contributor to the development and the maintenance of the GBM-podocyte interaction.

Urinary Int*α*3 has been examined to determine if it can be utilized in the clinical setting as a biomarker of podocyte injury. Data from an investigation examining Int*α*3 as a urinary biomarker for detecting the presence of varying glomerular diseases concluded that it had a diagnostic sensitivity of 18.6% and specificity of 100% [57]. The data from this one reported study and a lack of any other investigations do not support the utilization of Int*α*3 as a diagnostic urinary biomarker for glomerulopathies.

## 11. CD59

CD59 (also referred to as protectin or 20 kDa homologous restriction factor—HRF20) is a glycosylphosphatidylinositol-anchored membrane bound protein that regulates the formation of the terminal complement complex of the immune complement cascade [128]. Regulation of the formation of the terminal complement complex is done so by the inhibition of the binding of C9 to the C5b–C8 complex. In the developing kidney, CD59 protein and mRNA are expressed in the ureteric duct epithelium, while in the mature, adult kidney expression they are found in the collecting ducts [96]. CD59 immunofluorescent expression is also notable in frozen sections of renal cortex—specifically in podocytes, tubular epithelium, and endothelial cells—and in cultured podocytes [129]. The role of CD59 in renal development is not clear, though it may act through inhibition of formation of the terminal complement complexes which could otherwise attenuate normal cellular growth. Regulation of the complement cascade within the glomerulus by CD59 is suggested by its ability to inhibit the complement mediated immune response to subepithelial immune complex deposition [130].

CD59 has not been utilized widely as a biomarker of podocyte injury, but a single study did present data supporting further investigations. When the CD59 immunofluorescent expression in renal tissue samples from patients diagnosed with membranous glomerulonephritis was compared to that in healthy controls, membranous glomerulonephritis patients had lower CD59 expression [131]. CD59 concentration in the urine of patients with membranous glomerulonephritis was higher in comparison to urinary CD59 from healthy controls and patients with diabetic nephropathy. The same investigation presented that urinary CD59 levels did not correlate to serum creatinine, urinary protein concentration, or duration of disease. With a paucity of data in hand, conclusions on CD59's usefulness as a histologic or urinary biomarker of podocyte injury are constrained.

## 12. Wilms Tumor Protein 1 (WT1)

A podocyte transcription factor—WT1—can been found in urine (associated with cells or exosomes) and as thus is a novel biomarker for recognizing podocyte injury. As any other typical transcription factor, WT1 activity is upregulated by certain stimuli, and its activity is responsible for the promotion of selective gene expression. WT1 regulates the expression of *Nphs1* and *Podxl* in mice, as both transcripts are reduced in *Wt1* knockouts [132]. In the same *Wt1* knockout mice, crescentic glomerulonephritis or mesangial sclerosis can be observed. WT1 has been shown to directly bind to the *Podxl* gene promoter, and through this mechanism it influences expression [133]. WT1 is first noted in the C- and S-shaped glomerular bodies of the developing kidney, and in the mature kidney, WT1 can be found in glomerular podocytes and arteriole endothelium [111]. *WT1* is mutated in patients with the Denys-Drash syndrome and the Frasier syndrome, both which are present with nephrotic syndrome and extra-renal malformations within the genital urinary system [132]. It should be noted that while WT1 is a proven histologic biomarker of podocytes [4, 118, 134], in a number of glomerulopathies WT1 protein is immunohistochemically expressed in glomerular parietal epithelium; therefore, WT1 as a biomarker of podocytes may not be exclusive and specific in diseased states [4, 135].

Urinary WT1 protein as a biomarker of podocyte injury has been described in one study, and thus this is an initial presentation of its utility. Data showed that by utilizing a passive Heymann nephritis model, WT1 protein and *Wt1* mRNA could be quantified from cells isolated from collected urine samples [46]. Unfortunately with only one study in hand, not much can be made in terms of deduction on the efficacy of WT1 protein as a biomarker of podocyte injury; however, investigation of WT1 from urinary exosomes shall be discussed in the following section.

## 13. Podocyte Specific mRNA

Podocyte specific mRNA has been detected in urine, and its usefulness as a biomarker of podocytopathy has been described. Nephrin, podocin, synaptopodin, podocalyxin, *CD2AP*, *ACTN4* (encodes for *α*-actinin 4), *PTPRO* (encodes for GLEPP-1), and *WT1* mRNA have been isolated from urine of patients with varying glomerulopathies. Review of urinary nephrin, podocin, synaptopodin, podocalyxin, *B7-1,* and *PTPRO* mRNA has already been described and can be found in their respective sections above. *CD2AP*, *ACTN4*, and *WT1* urinary mRNA shall be discussed as follows.

Urinary WT1 protein and *Wt1* mRNA expression were observed and analyzed from cells isolated from urine samples of passive Heymann nephritic rats, and there was a concomitant decrease in glomerular podocyte quantity as proteinuria worsened [46]. This data would lend to the hypothesis that glomerular podocytes in certain glomerulopathies are shed and lost in the urine, and those podocytes in urine could directly relate to the severity of podocyte/glomerular injury. *CD2AP* and *WT1* mRNA were found in urinary derived podocytes of both FSGS patients and healthy humans; therefore, quantitative analysis should be recommended in determining if disease is present [45]. Urinary *WT1* and *ACTN4* mRNA could also be collected from some dogs and cats with chronic kidney disease, but this study did not contain healthy control groups, so the significance of these findings has yet to be determined [95]. Urinary *CD2AP* and *ACTN4* mRNA expression were significantly increased in patients with diabetic nephropathy compared to healthy controls, the level of mRNA increased with progression of disease, and the levels of urinary *CD2AP* and *ACTN4* mRNA positively correlated with levels of urinary albumin excretion, BUN, and with serum creatinine [69]. With the data presented in this and previous sections, along with the fact that very little sample is necessary for the quantification of mRNA transcript, there is promising evidence supporting podocyte specific urinary mRNA as a biomarker of injury.

## 14. Exosomal Transcription Factor WT1

Exosomes are vesicles that can be attained from urine via differential centrifugation, and they are excreted through the length of the nephron, including from podocytes. Generally they are found to be 50–100 nm in diameter and have been seen to contain a number of cytosolic and membrane proteins as shown with proteomic profiling [136]. The origin of exosomes can be described as such: endocytic vesicles fuse with multivesicular bodies, and thereafter multivesicular bodies can undergo invagination—taking into the new vesicles matter that may be within the cytosolic milieu (e.g., WT1). At this stage, there is a multivesicular body that contains vesicles, and at some point the multivesicular body can fuse with the cellular plasma membrane and exocytose the vesicles contained within it. Methods of urinary exosome preparation and storage have been fine-tuned so that under proper conditions the protein profile of exosomes remains intact over time for analysis [137].

Data from one study presented that podocyte specific urinary exosomes could be quantified from patients and animal models of glomerulopathies: the podocyte specific aspect of these exosomes as being the transcription factor WT1 [138]. In animal models for FSGS and collapsing glomerulopathy, urinary exosomal WT1 quantity significantly increased after insult, and in control animals urinary exosomal WT1 was undetectable. Likewise, in human patients with diagnosed FSGS urinary exosomal WT1 could be identified, while exosomal WT1 couldnot be detected in the urine of healthy volunteers. Thus, with the given data, urinary exosomal WT1 is specific for a diagnosis of glomerulopathy; however, more work will have to be done to elaborate on the usefulness of urinary exosomal WT1 as a biomarker of podocyte injury.

## 15. Podocalyxin Positive Granular Structures (PPGS)

Under microscopic examination of urine, granular structures can be identified, and if they are immunoreactive for the podocyte specific antigen podocalyxin are termed PPGS. PPGS have been proven to originate from podocytes via podocalyxin immunostaining and are thought to form through a process of tip vesiculation from microvillus formations of injured podocytes. PPGS can be differentiated from podocyte exosomes on three points: PPGS are found not to contain exosomal specific markers (CD24 and CD63), PPGS are larger than 100 nm in diameter (exosomes are typically smaller), and in kidney samples from patients with PPGS found in urine, histologic evidence of exocytosis—the mechanism of by which exosomes are excreted—is not prevalent [103, 139]. While PPGS are immunoreactive for podocalyxin, there is weak staining against other podocyte apical membrane specific proteins (GLEPP-1 and CR1), and there was no observed staining against slit diaphragm specific ZO-1 and podocyte basal membrane specific Int*α*3 [103]. This further supports that PPGS originate from the apical membrane of podocytes. It is also notable that urinary supernatant has greater amount of podocalyxin protein than urinary sediment (this is true for urine of both healthy and patients with diagnosed glomerulopathies) and that within the sediment PPGS are found within casts. Therefore, quantification of PPGS from supernatant would be of higher yield than from centrifuged urinary sediment. When urine of patients with active glomerulonephritis and nephrotic syndrome was compared to that from healthy controls, greater amount of excreted PPGS could be observed in the urine of diseased patients [139]. Healthy controls excreted low levels of PPGS, and it may be explained by normal turnover of podocytes. In glomerulonephritis patients (diagnosed with IgA nephropathy, Henoch-Schönlein purpura nephritis, or lupus nephritis) and patients with a diagnosis of nephrotic syndrome, urinary sedimentary podocalyxin (which PPGS was a constituent) was greater than in healthy controls, and podocalyxin concentration was significantly greater in the urinary sediment of glomerulonephritis patients than that of the nephrotic patients [103]. PPGS could also be observed in the urine of glomerulonephritis patients that did not exhibit podocyturia, therefore showing that PPGS may be more sensitive in detecting glomerulopathy and podocyte injury [57]. The described data shows that PPGS are a more focused biomarker than general podocalyxin in the urine—keeping in mind that podocalyxin may be present in the urine additionally in association with whole podocytes. Utilization of PPGS as a biomarker of podocyte injury seems promising; however, as with all of the discussed biomarkers above, thorough clinical studies it would be needed to strengthen the argument for its clinical usage.

## 16. Conclusion

It has been found that podocyte injury can be diagnosed and monitored through the utilization of urinary derived biomarkers. The findings that podocalyxin and nephrin proteins can be detected in the urine of a significant percentage of normoalbuminuric, diabetic patients highlight the screening utility that these two podocyte specific proteins carry. Since monitoring urinary albumin potentially results in the passing over of a significant segment of diabetic patients—a fraction of today's population that cannot be dismissed—the medical field may wish to examine closely the earlier detection of diabetic nephropathy garnered from utilizing a podocyte specific marker. One could also monitor glomerulopathy progression with a urinary derived biomarker, diverting the need for follow-up renal biopsy if the patient was not physiologically stable or did not desire for such a procedure. Monitoring urinary biomarkers of podocyte injury also holds the capacity to be a cheaper method of diagnosis than the renal biopsy—a concept that we cannot ignore as the financial environment pressures the budget of healthcare. With respect to other biomarkers used in clinical practice, urinary biomarkers could be utilized in such manners as supplemental evidence of disease (as troponins are used alongside electrocardiography to aid in the diagnosis of myocardial infarction) or for the monitoring of disease progression with analysis of therapeutic efficacy (as hemoglobin A1C is used in patients with diabetes mellitus). The strength of urinary podocyte biomarkers in clinical utility would be measured the best in their combination with the clinical picture of the patient and other determinants of renal function, and they most likely will not themselves usurp the quantification of proteinuria as a measure of glomerular function. The utilization of the podocyte specific urinary biomarkers has not yet translated into common clinical practice, while the age-old quantification of proteinuria and renal biopsy are the mainstays that clinicians have at hand and will probably continue to rely on. However, with further investigations of the podocyte biomarkers discussed this stance could change, and attest to their utility. The reader is referred to Table 1 as a synoptic of the discussed urinary biomarkers and their prognostic correlation to specifically studied glomerulopathies.

By definition, a biomarker is any parameter of a patient/animal model that can be quantified, and through its quantification a normal physiologic state, a pathologic state, or disease response to initiated therapy can be indicated [140]. Before any of the described urinary biomarkers of podocyte injury can be used in the clinical setting, they should express a number of characteristics. Characteristics of a renal biomarker satisfactory for regular usage would include the following: that its quantification is simple, accurate, and reproducible; it is a sensitive indicator of kidney injury or a sensitive indicator of disease response to treatment; it gives clinicians a budget-sensitive tool; it provides the clinician additional information not yet attained by other tests/examinations; it diagnoses and differentiates between renal pathologies; it is clinically applicable in differing demographics [38, 141]. Since the classic monitoring of proteinuria/albuminuria does not meet all of the described characteristics of an ideal biomarker, possibly the novel biomarkers in this paper may complement data gleaned from such conventional tests and provide the clinician more information on the presence of a glomerulopathy.

It is also obvious from this review of the literature that more work will be needed to determine if the discussed urinary biomarkers of podocyte injury are suitable for clinical use. In addition for the need for further investigation of the already mentioned biomarkers, there are other possible podocyte specific biomarkers that could be quantified in urine. For example, neurexin-1 is a slit diaphragm protein that also has indirect interaction with the podocyte actin cytoskeleton and therefore may be influential in regulating both podocyte function and architecture [142, 143]. Another potential candidate for usage as a urinary biomarker of podocyte injury is myosin heavy chain 9 (MYH9), which is a subunit of a larger protein named nonmuscle myosin IIA [8]. Mutation in the gene that encodes for MYH9 is found to be responsible for Fechtner's syndrome, and in addition, MYH9 has been identified in urine samples [136, 144]. There are potentially other components of podocytes that could be identified in the urine of patients afflicted with glomerulopathies useful as biomarkers, and as such this provides a number of possible routes of investigation to follow.

## Figures and Tables

**Table 1:** Urinary biomarkers of podocytopathy in patients: increased urinary levels correlate with the presence or the advancement of glomerular disease (except when otherwise noted*).

Protein		mRNA		Exosomal transcription factor		PPGS
Podocalyxin	IgAN [55, 56, 60, 61] HSPN [55, 56] DN [58, 67] FSGS—increased u-podocalyxin correlates with better prognosis [62]* AKI—increased u-podocalyxin in recovering patients [66]* PE [65]	Podocalyxin	DN [69]	WT1	FSGS [138]	AcGN [139] NS [103, 139] IgAN [103] HSPN [103] LN [103]

Nephrin	DN [77, 78] PE [65, 80]	Nephrin	DN [87, 89] PE [91] FSGS [90] LN [86, 88] IgAN [89] MCD [89] MN [89]

Podocin	PE [64]	Podocin	PE [91] DN [69, 89] IgAN [89] MCD [89] MN [89] LN [85] and—decrease in u-podocin mRNA follows decrease in GFR [88]*

CR1	AuGN [103] NS [103] LN [57] and—lower u-CR1 in advanced cases [102]* MN [57] MPGN [57] AS [57] PSAGN [57] HSPN [57] IgAN [57]	*CD2AP *	DN [69]

CD80	Relapsed MCD [104]	*B7*-*1* ^a^	FSGS [90]

Mindin	DN [123]	Synaptopodin	DN [69, 87, 113] LN—decrease of u-synaptopodin mRNA follows decrease in GFR [88]*

CD59	MG [131]	*AC* *TN*4^b^	DN [69]

Numbers in parentheses refer to citations that can be noted in the References section of this paper.

Abbreviations: messenger ribonucleic acid (mRNA), complement receptor 1 (CR1), Wilms tumor protein 1 (WT1), CD-2-associated protein (CD2AP), podocalyxin positive granular structures (PPGS), urinary podocalyxin (u-podocalyxin), urinary CR1 (u-CR1), urinary podocin mRNA (u-podocin mRNA), urinary synaptopodin mRNA (u-synaptopodin mRNA), IgA nephropathy (IgAN), Henoch-Schönlein purpura nephritis (HSPN), diabetic nephropathy (DN), focal segmental glomerulosclerosis (FSGS), acute kidney injury (AKI), preeclampsia (PE), lupus nephritis (LN), minimal change disease (MCD), membranous nephropathy (MN), nephrotic syndrome (NS), acute glomerulonephritis (AuGN), active glomerulonephritis (AcGN), membranoproliferative glomerulonephritis (MPGN), Alport syndrome (AS), and poststreptococcal acute glomerulonephritis (PSAGN).

*This table summarizes that the majority of studies presented direct correlation of increased urinary levels of a biomarker with the incident or worsening of a specific glomerular disease; however, some studies (as noted with an asterisk and a short explanation) presented opposite findings and are thus highlighted.

^a^ *B7-1* encodes for CD80.

^b^ *ACTN4* encodes for *α*-actinin 4.
